# *ORC6* drives the malignant progression of cervical squamous intraepithelial lesions and serves as a biomarker for high-grade dysplasia

**DOI:** 10.3389/fonc.2026.1795793

**Published:** 2026-05-29

**Authors:** Rouyi Chen, Lu Huang, Yun Huang, Feihu Chen, Lijun Zhong, Fanyan Ou, Yang Yang, Yinghui Wu

**Affiliations:** Department of Pathology, The First People’s Hospital of Yulin, Yulin, China

**Keywords:** cell cycle, cervical cancer, HPV-related cervical lesions, immune infiltration, Orc6

## Abstract

**Background:**

Origin Recognition Complex Subunit 6 (*ORC6*) is essential for DNA replication initiation. However, its dynamic expression patterns and potential biological functions during the progression of cervical lesions remain unclear.

**Methods:**

We analyzed the differential expression of *ORC6* in cervical cancer and normal tissues using an integrated TCGA-GTEx-CESC dataset and explored its potential regulatory mechanisms via GO/KEGG, GSEA, and CIBERSORT immune infiltration analyses. Furthermore, a total of 163 clinical tissue specimens (including CIN I, CIN II, CIN III, and invasive cervical carcinoma) were examined by immunohistochemistry (IHC) to evaluate *ORC6* protein expression and its association with pathological grade and clinicopathological characteristics.

**Results:**

Bioinformatics analysis revealed that *ORC6* was significantly upregulated in cervical cancer (*P* < 0.001) and was associated with transcriptional programs related to E2F targets, DNA replication, and the G2/M checkpoint. Immune infiltration analysis showed that elevated *ORC6* expression was associated with a distinct immune infiltration pattern, characterized by increased M0 macrophages and reduced activated NK cells, resting NK cells, and CD8+ T cells. IHC analysis further demonstrated that *ORC6* expression was significantly increased in cervical lesions and invasive carcinoma compared with normal cervical tissues (*P* < 0.001). Notably, *ORC6* expression was significantly higher in the CIN II–III group than in the CIN I group (*P* < 0.01). In contrast, *ORC6* expression was not significantly associated with tumor differentiation, lymph node metastasis, vascular invasion, perineural invasion, or overall survival (all *P* > 0.05).

**Conclusion:**

*ORC6* is closely associated with the progression of HPV-related cervical lesions, particularly the transition from CIN I to CIN II–III. *ORC6* may serve as a potential auxiliary biomarker for the identification and early stratification of high-grade squamous intraepithelial lesions (HSIL).

## 1 Introduction

Cervical cancer remains one of the most prevalent malignancies affecting women worldwide, ranking fourth in both incidence and mortality among female cancers. Despite the significant reduction in incidence achieved through widespread vaccination and human papillomavirus (HPV) screening, it continues to be a leading cause of cancer-related death, particularly in low- and middle-income countries (1). Persistent infection with high-risk HPV (HR-HPV) has been established as a necessary etiological prerequisite for cervical carcinogenesis, with HPV16 accounting for approximately 60% of all cases. The progression from initial HPV infection to invasive cervical cancer (2) is a multi-stage, stepwise pathological evolution, typically advancing from low-grade squamous intraepithelial lesions (LSIL/CIN I) to high-grade squamous intraepithelial lesions (HSIL/CIN II-III), and ultimately to invasive carcinoma (3). Therefore, a profound understanding of the molecular mechanisms underlying this progression is imperative for achieving precise early screening and effectively intercepting malignant transformation.

In recent years, the investigation and application of molecular biomarkers have significantly propelled the precision diagnosis and treatment of cervical cancer. For instance, the dual staining of p16 and Ki-67 has become a standard tool for auxiliary pathological diagnosis, while HR-HPV DNA testing has markedly improved the sensitivity of screening for precancerous lesions (4). However, existing biomarkers primarily focus on viral infection status or cell proliferation levels, and do not fully reflect the intrinsic molecular mechanisms driving the malignant progression of cervical lesions. Consequently, there is an urgent clinical need to identify novel biomarkers that can accurately reflect the malignant potential of cervical lesions to further optimize early screening and risk stratification strategies.

The Origin Recognition Complex (ORC) is a six-subunit DNA-binding complex that orchestrates the initiation of DNA replication and the assembly of the pre-replication complex (pre-RC) in eukaryotic cells (5). The core mechanism by which HPV oncoproteins E6 and E7 drive cervical carcinogenesis involves the inactivation of the p53 and Rb pathways, leading to the release of E2F transcription factors, which in turn forcibly initiates the cell cycle and DNA replication (6). As a core component of the ORC family, *ORC6* not only initiates DNA replication in response to E2F signaling but also possesses non-replicative functions in regulating chromosome segregation and cytokinesis (7–9). Multi-omics pan-cancer studies have revealed that *ORC6* is upregulated in the majority of tumor types and correlates with advanced clinical stages and poor prognosis. Furthermore, *ORC6* expression exhibits a specific correlation with various immunosuppression-related genes (e.g., PD-L1/CD274, TGFBR1) and is associated with the infiltration of regulatory T cells in certain tumors (10, 11), suggesting its potential role in reshaping the tumor immune microenvironment (TIME) and facilitating immune evasion. Nevertheless, the expression characteristics and biological functions of *ORC6* specifically during the progression of cervical lesions remain unclear. Based on these premises, this study systematically analyzed the expression patterns, potential biological pathways, and immune infiltration profiles of *ORC6* in cervical cancer by integrating transcriptomic data from the TCGA database. Concurrently, we validated our findings using immunohistochemistry (IHC) on a clinical cohort of 163 tissue samples comprising different grades of CIN and invasive cancer. Our study aims to elucidate the dynamic expression changes of *ORC6* during the progression of cervical squamous intraepithelial lesions and to explore its clinical value as a biomarker for auxiliary CIN grading and early warning of malignant transformation.

## 2 Materials and methods

### 2.1 Data acquisition and preprocessing for bioinformatics analysis

Transcriptomic data for this study were obtained from the UCSC Xena database. The integrated TCGA-GTEx-CESC dataset included expression profile data from The Cancer Genome Atlas-Cervical Squamous Cell Carcinoma and Endocervical Adenocarcinoma (TCGA-CESC) project and the Genotype-Tissue Expression (GTEx) project. After preprocessing and sample matching, a total of 298 cervical cancer samples and 13 normal cervical tissue samples were included in the final analysis, including 3 normal samples from TCGA-CESC and 10 normal cervical tissue samples from GTEx. Differential expression analysis was performed using the DESeq2 package, with screening thresholds set at |log_2_ FoldChange| > 1 and an adjusted P value (*P*adj*)* < 0.05. Volcano plots were generated using the ggplot2 package to visualize upregulated, downregulated, and non-significant genes. Heatmaps of *ORC6* and representative associated genes were constructed using the pheatmap package, with expression levels normalized as Z-scores.

### 2.2 Functional enrichment analysis and PPI network construction

TCGA tumor samples were classified into *ORC6*-high and *ORC6*-low groups using the median *ORC6* expression value as the cutoff (4.04 log_2_(TPM + 1)), equivalent to 15.41 TPM). Differentially expressed genes (DEGs) between the two groups were identified and used for downstream analyses. Gene Ontology (GO) functional annotation and Kyoto Encyclopedia of Genes and Genomes (KEGG) pathway enrichment analyses were performed on *ORC6*-related DEGs using the clusterProfiler package. To explore the potential molecular mechanisms underlying *ORC6*, Gene Set Enrichment Analysis (GSEA) was conducted using the hallmarks gene sets (h.all.v7.4.symbols.gmt) from the MSigDB database. The number of permutations was set to 1000, and significance was defined as *P*adj < 0.05 and FDR < 0.25. Furthermore, a Protein-Protein Interaction (PPI) network for *ORC6*-related DEGs was constructed using the STRING database and visualized using Cytoscape software.

### 2.3 Immune infiltration analysis

Immune cell infiltration was estimated using the CIBERSORT algorithm with the LM22 signature matrix, which deconvolutes bulk RNA-seq expression profiles into 22 immune cell types. Gene expression data (TPM-normalized) were used as input without quantile normalization (QN = FALSE). Statistical significance of deconvolution results was assessed by 500 permutation tests, and samples with a Benjamini-Hochberg (BH) false discovery rate (FDR) < 0.05 were retained for downstream analysis, resulting in 149 samples (Normal n=7, Tumor n=142). Differences in immune cell proportions between Normal and Tumor groups were evaluated using the Wilcoxon rank-sum test, followed by BH correction for multiple comparisons. The correlation between *ORC6* expression and immune cell infiltration levels was assessed using Spearman correlation analysis. All analyses and visualizations were performed in R (version 4.5.0).

### 2.4 Clinical sample collection

A total of 163 paraffin-embedded cervical lesion specimens were retrospectively collected from the Department of Pathology at Yulin First People’s Hospital between January 2022 and December 2024. The cohort included 34 cases of LSIL/CIN I, 59 cases of HSIL (including CIN II and CIN III), and 70 cases of invasive cervical carcinoma (ICC). All diagnoses were reviewed and confirmed by two senior pathologists. None of the patients received preoperative chemotherapy or radiotherapy. This study was approved by the Medical Ethics Committee of Yulin First People’s Hospital (Approval No. YLSY-IRB-SR-2023005).

### 2.5 Immunohistochemistry staining and evaluation

*ORC6* protein expression was detected using the MaxVision two-step IHC method. Sections were incubated with anti-*ORC6* antibody (1:800 dilution; Cat. No. ab153993, Abcam, Shanghai, China) following antigen retrieval with EDTA buffer, strictly according to the manufacturer’s instructions. The results were evaluated independently by two pathologists in a double-blind manner. A semi-quantitative Immunoreactive Score (IRS) system was adopted for assessment: (1)Staining Intensity Score:0(negative), 1 (pale yellow/weak), 2 (brownish-yellow/moderate), and 3 (dark brown/strong). (2)Percentage of Positive Cells Score: 0 (<5%), 1 (5%–25%), 2 (26%–50%), 3 (51%–75%), and 4 (>75%). (3) Final Score: Calculated by multiplying the intensity score by the percentage score, resulting in a total score ranging from 0 to 12. Samples were classified into high- or low-expression groups based on the median IRS score.

### 2.6 Statistical analysis

Statistical analyses were performed using SPSS 26.0 (IBM Corp., Armonk, NY, USA) and GraphPad Prism 9.0 (GraphPad Software, San Diego, CA, USA). The Chi-square (X^2^) test or Fisher’s exact test was used to analyze the relationship between *ORC6* protein expression and the clinicopathological characteristics of patients. Differences in *ORC6* IHC scores among the CIN I, CIN II-III, and ICC groups were analyzed using the Kruskal-Wallis H test (for multiple groups) or the Mann-Whitney U test (for two groups). Survival curves were generated using the Kaplan-Meier method, and the Log-rank test was used to compare the overall survival (OS) between the *ORC6* high- and low-expression groups in the TCGA cohort. A two-tailed *P-value* < 0.05 was considered statistically significant.

## 3 Results

### 3.1 *ORC6* is upregulated in cervical cancer

**Figure 1:**
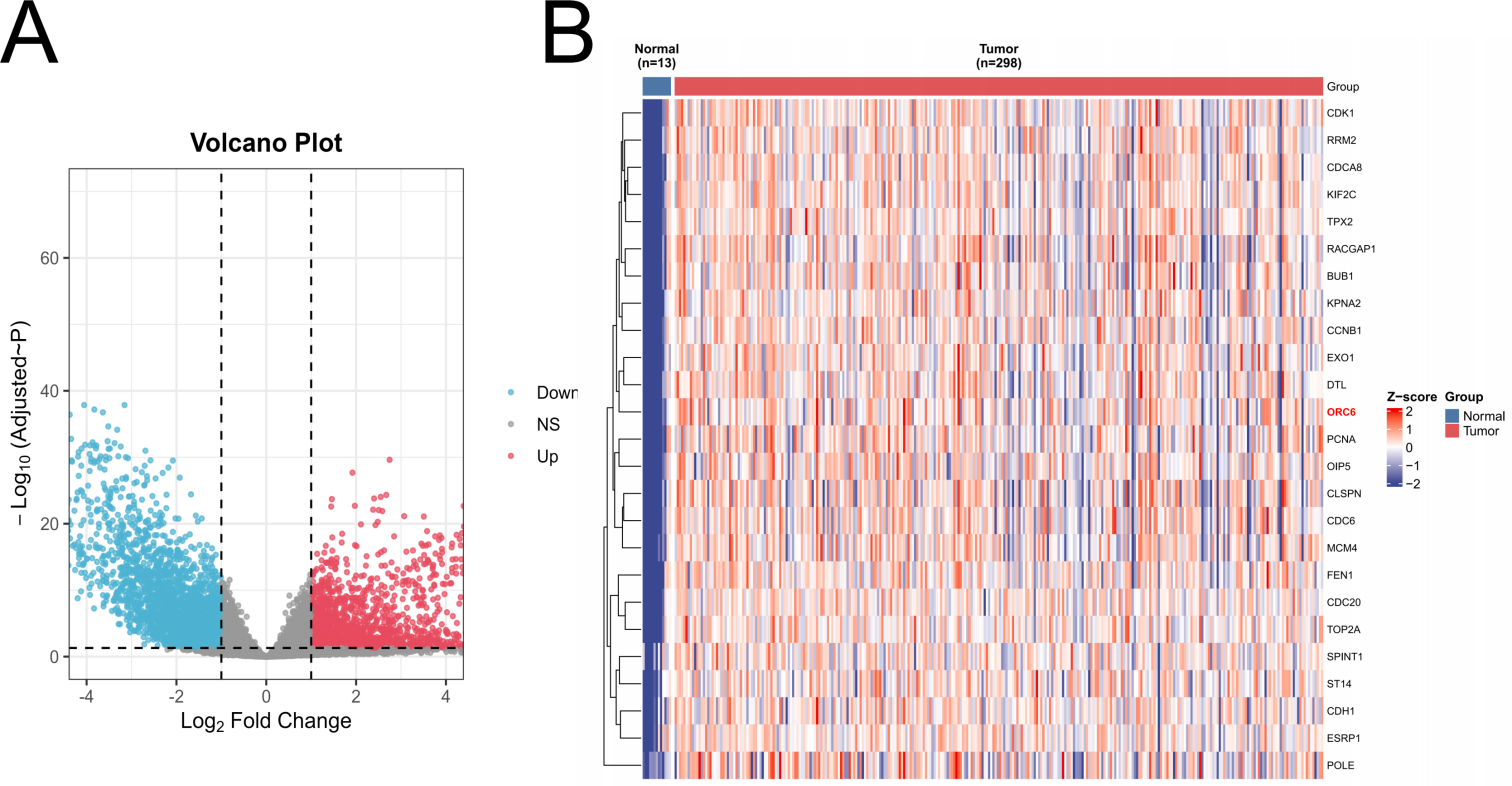
Differential expression analysis. **(A)** volcano plot of differentially expressed genes in cervical cancer tissues (n = 298) versus normal cervical tissues (n = 13). Red dots indicate significantly upregulated genes, blue dots indicate significantly downregulated genes, and gray dots indicate non-significant genes. **(B)** heatmap showing the normalized expression patterns of *ORC6* and representative genes in normal cervical tissues (n = 13) and cervical cancer tissues (n = 298). Columns represent individual samples, and rows represent genes. *ORC6* is highlighted in red.

Analysis of the combined TCGA-CESC and GTEx dataset revealed that *ORC6* expression levels were significantly elevated in cervical cancer tissues compared to normal cervical tissues (Normal n=13, Tumor n=298, P < 0.001). Differential expression analysis identified a total of 818 differentially expressed genes (DEGs), comprising 127 upregulated and 691 downregulated genes (|log_2_FC| > 1, BH-adjusted P < 0.05). The distribution of these DEGs is illustrated in the volcano plot (**Figure 1A**), where red dots represent significantly upregulated genes and blue dots represent significantly downregulated genes. *ORC6* was classified within the significantly upregulated gene set (log_2_FC=3.14, *P*adj = 1.66×10^-15^). A heatmap of *ORC6* and representative genes further illustrated the distinct expression pattern between normal and tumor tissues (**Figure 1B**). *ORC6* showed a higher expression pattern in tumor tissues than in normal tissues.

### 3.2 *ORC6*-associated gene expression patterns and PPI network analysis

**Figure 2:**
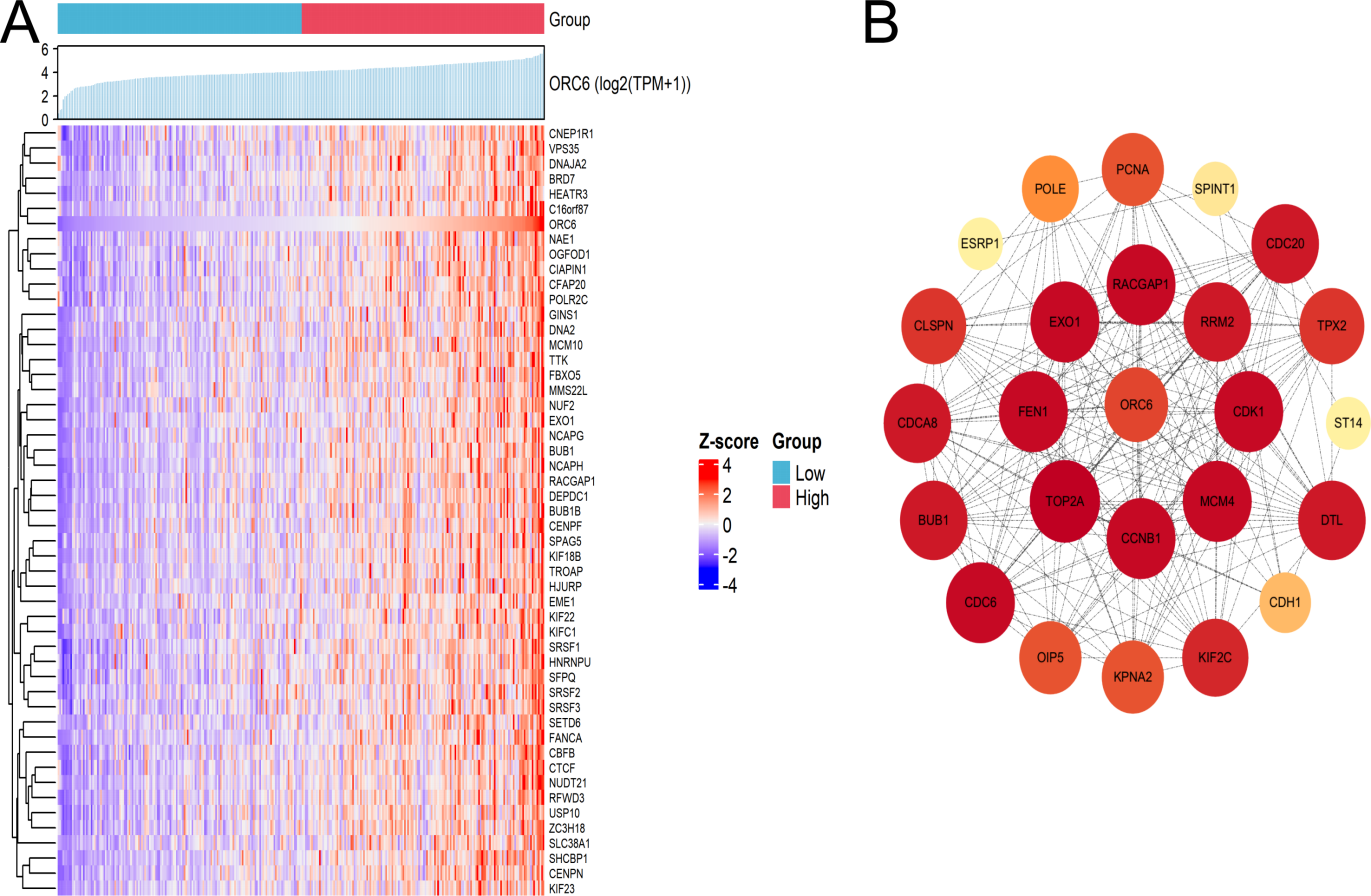
**(A)** heatmap showing the normalized expression patterns of representative genes in the *ORC6*-high and *ORC6*-low groups. **(B)** PPI, protein-protein interaction network of *ORC6* and representative *ORC6*-associated genes generated using the STRING database. Nodes represent proteins, and edges represent predicted or known interactions.

Based on the median *ORC6* expression value in the TCGA tumor cohort (4.04 log_2_(TPM + 1), equivalent to 15.41 TPM), cervical cancer samples were divided into *ORC6*-high and *ORC6*-low groups for differential expression analysis. As shown in **Figure 2A**, distinct differences in gene expression patterns were observed between the two groups. Concomitant with the upregulation of *ORC6*, a cluster of genes closely associated with cell proliferation exhibited a significant co-upregulation trend, including *MCM10*, *TTK*, *EXO1*, *NUF2*, *BUB1*, *CENPF*, and *KIF23*. These genes are known to participate in DNA replication, chromosome segregation, and mitotic processes (12–15). Furthermore, the constructed Protein-Protein Interaction (PPI) network of *ORC6* and its co-expressed genes revealed that *ORC6* forms a dense interaction network with multiple hub proteins of the cell cycle, such as *MCM4, TOP2A, CCNB1, CDK1, CDC6, RACGAP1*, and *KIF2C* (**Figure 2B**). Collectively, these findings suggest that elevated *ORC6* expression is accompanied by coordinated transcriptional changes in genes involved in cell-cycle progression, DNA replication, and mitosis in cervical cancer.

### 3.3 Functional enrichment analysis of *ORC6*-associated genes

**Figure 3:**
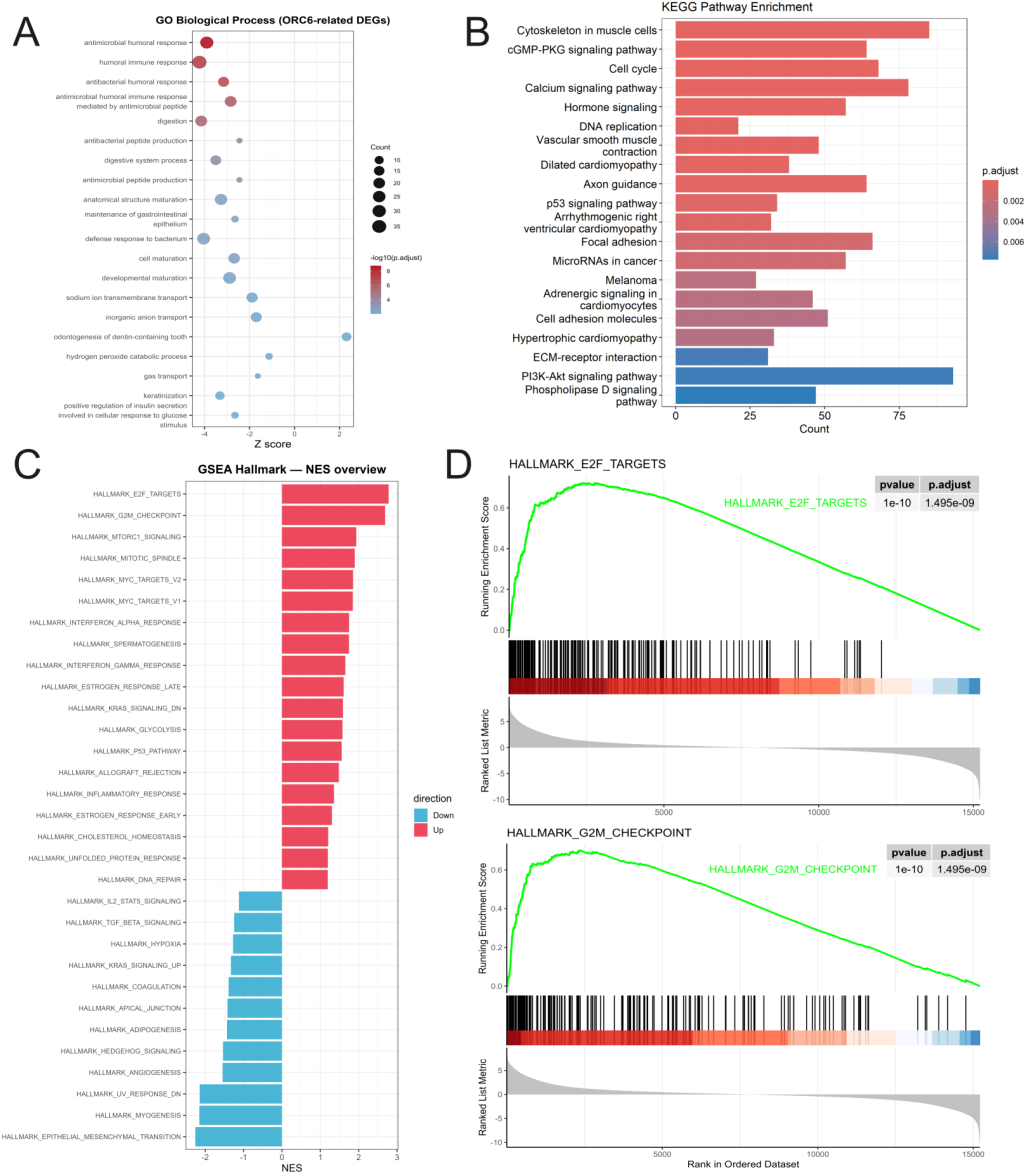
Functional enrichment and pathway analysis of *ORC6*-related genes in cervical cancer. **(A)** GO, gene ontology biological process enrichment analysis of differentially expressed genes associated with *ORC6*. **(B)** KEGG, Kyoto encyclopedia of genes and genomes pathway enrichment analysis. **(C)** GSEA, gene set Enrichment Analysis based on hallmark gene sets comparing *ORC6* high- and low-expression groups. **(D)** representative enrichment plots for the E2F targets and G2M checkpoint pathways.

To further investigate the potential biological functions of *ORC6* in cervical cancer, we performed GO, KEGG, and GSEA functional enrichment analyses using the differentially expressed genes (DEGs) identified between the *ORC6*-high and *ORC6*-low groups. Gene Ontology (GO) biological process enrichment results showed that these genes were primarily involved in humoral immune response, antimicrobial humoral response, and maintenance of epithelium (**Figure 3A****).** These findings suggest that differences in *ORC6* expression are accompanied by transcriptomic changes related to both epithelial homeostasis and immune-associated biological processes.

KEGG pathway enrichment analysis further revealed that, in addition to being enriched in pathways such as cell cycle, DNA replication, and the p53 signaling pathway, as well as several immune-related pathways (**Figure 3B**). These results indicate that the transcriptional changes associated with high *ORC6* expression are linked to pathways involved in cell-cycle regulation, DNA replication, and stress-response signaling.

To further evaluate pathway-level differences between the *ORC6*-high and *ORC6*-low groups, we performed Gene Set Enrichment Analysis (GSEA) using the ranked whole-transcriptome expression dataset. The results showed that classical gene sets related to cell proliferation and damage repair, such as *E2F* targets, *G2M* checkpoint, DNA repair, and mitotic spindle, were positively enriched in the *ORC6*-high group (NES > 0, **Figure 3C**). Among these, *E2F* targets and *G2M* checkpoint showed the strongest enrichment signals, as illustrated by the representative enrichment plots (**Figure 3D**). Overall, these enrichment analyses indicate that elevated *ORC6* expression is associated with transcriptional programs related to cell-cycle progression, DNA replication, and genome maintenance in cervical cancer.

### 3.4 Association of *ORC6* expression with immune cell infiltration in cervical cancer

**Figure 4:**
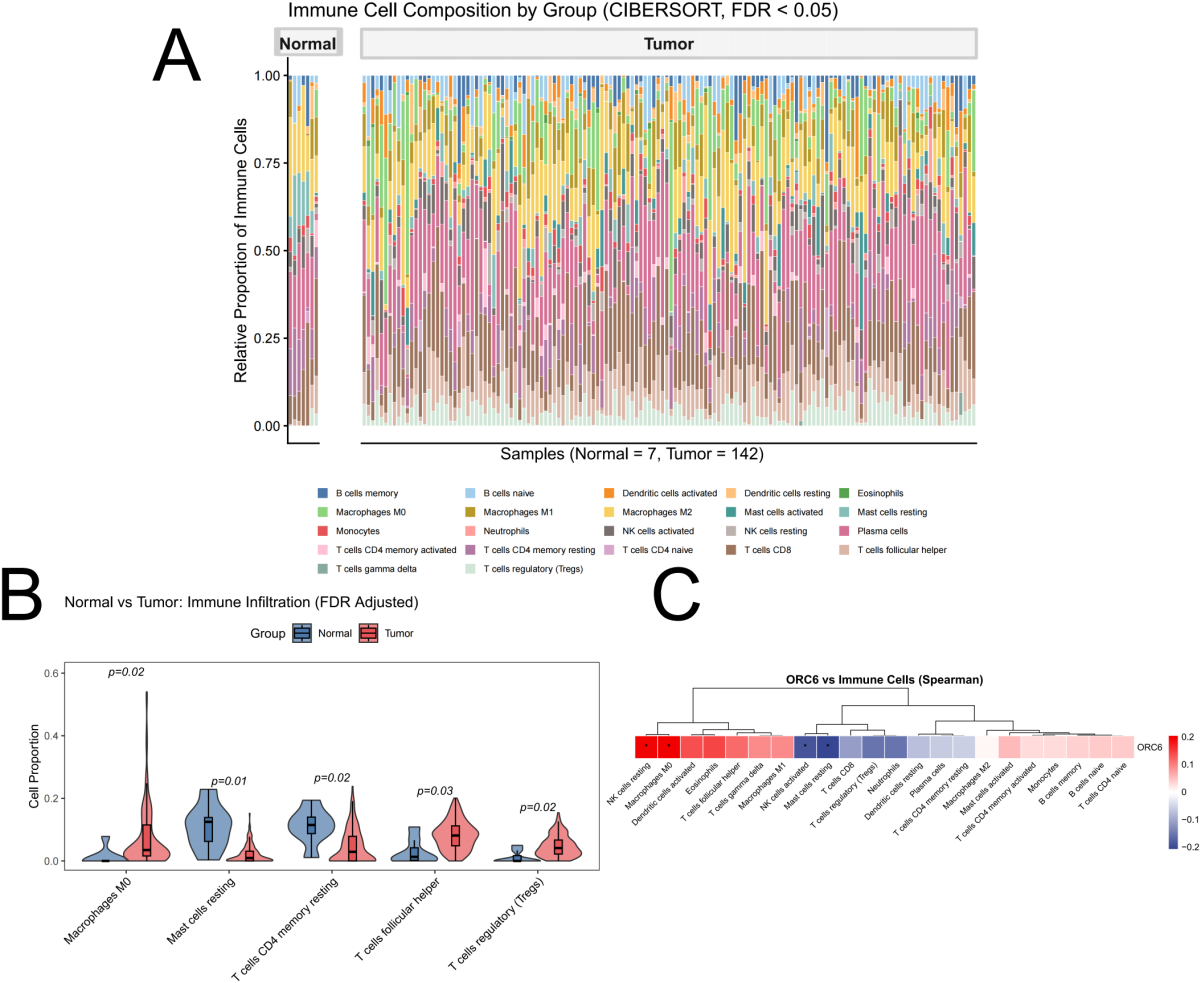
Immune cell infiltration landscape and association with *ORC6* expression in cervical cancer. **(A)** stacked bar chart showing the relative proportions of 22 immune cell types estimated by CIBERSORT in 7 normal cervical tissues and 142 tumor samples (FDR < 0.05). **(B)** violin plots of five immune cell types significantly altered between normal and tumor tissues after BH correction (all *P*adj < 0.05). **(C)** heatmap of Spearman correlation coefficients between *ORC6* expression and 22 immune cell types. Red and blue indicate positive and negative correlations, respectively.

To characterize the immune microenvironment of cervical cancer, we performed CIBERSORT-based immune cell deconvolution on 149 samples (7 normal cervical tissues and 142 tumor samples) that passed the CIBERSORT significance threshold (FDR < 0.05). The stacked bar plot revealed marked heterogeneity in immune cell composition across tumor samples, with notable differences in overall immune cell distribution compared to normal cervical tissues (**Figure 4A**). To identify immune cell types with differential infiltration between normal and tumor tissues, Wilcoxon rank-sum tests were performed followed by Benjamini-Hochberg (BH) correction for multiple comparisons across all 22 immune cell types. Five cell types showed statistically significant differences after FDR adjustment (all *P*adj < 0.05, **Figure 4B**): Macrophages M0, T cells follicular helper and T cells regulatory (Tregs) were enriched in tumor tissues, while Mast cells resting and T cells CD4 memory resting were significantly reduced compared to normal cervical tissues.

To further investigate the relationship between *ORC6* expression and the immune microenvironment, Spearman correlation analysis was performed between *ORC6* expression levels and the infiltration proportions of all 22 immune cell types. *ORC6* expression was positively correlated with NK cells resting and Macrophages M0 infiltration and negatively correlated with Mast cells resting and NK cells activated (all *P*adj < 0.05, **Figure 4C**). These findings indicate that elevated *ORC6* expression is associated with differences in immune cell infiltration patterns in cervical cancer.

### 3.5 Association of *ORC6* expression with overall survival in cervical cancer

**Figure 5:**
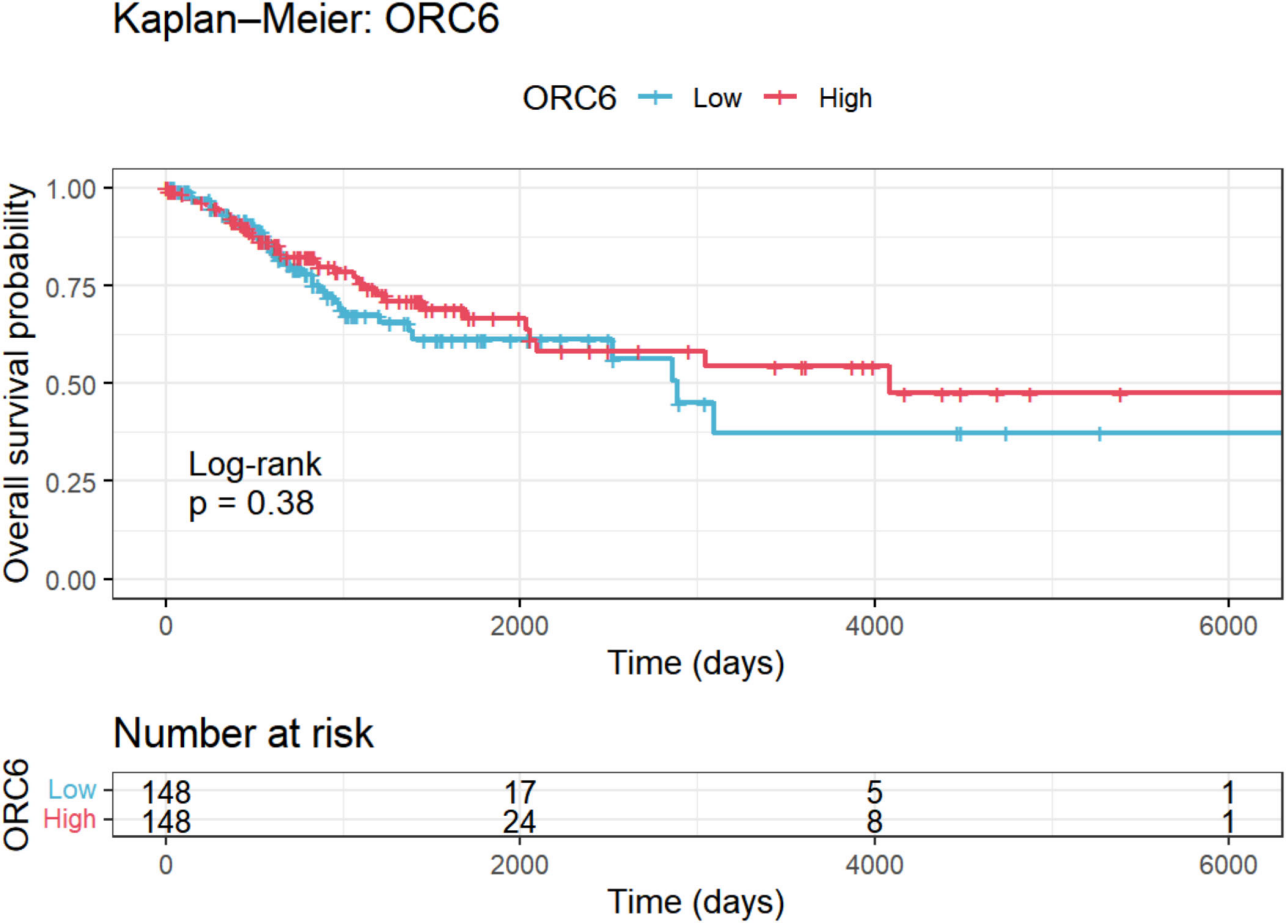
Kaplan-Meier survival analysis. Kaplan-Meier curves comparing OS, overall survival between *ORC6* high- and low-expression groups.

To assess the association between *ORC6* expression and survival outcome in cervical cancer, we performed Kaplan–Meier survival analysis using clinical follow-up data from the TCGA-CESC cohort. Patients were divided into high- and low-expression groups according to the median *ORC6* expression level. Although the high-*ORC6* group showed a numerically lower overall survival curve than the low-*ORC6* group, the difference was not statistically significant (log-rank test, P = 0.38; **Figure 5**).

### 3.6 Immunohistochemical expression patterns of *ORC6* in cervical lesions and its correlation with clinicopathological characteristics

**Figure 6:**
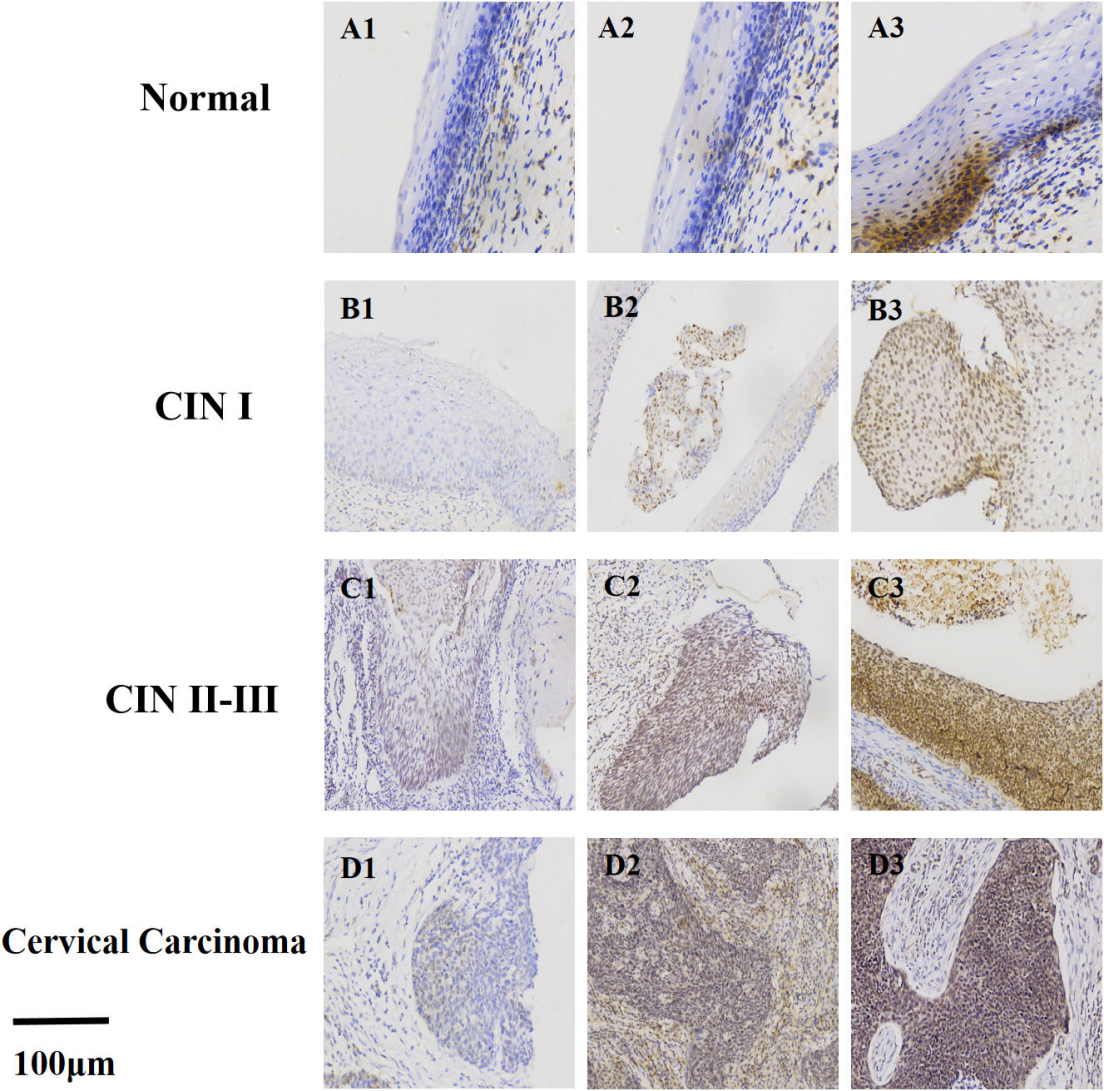
Immunohistochemical staining of *ORC6* protein expression in cervical tissue specimens. Representative immunohistochemical images showing *ORC6* protein expression in normal cervical epithelium **(A1–A3)**, cervical intraepithelial neoplasia grade I [CIN I, **(B1–B3)**], cervical intraepithelial neoplasia grade II–III (CIN II–III, **(C1–C3)**), and invasive cervical carcinoma **(D1–D3)**. *ORC6* expression was evaluated using the IRS, immunoreactive score system, calculated by multiplying staining intensity (0–3) by the percentage of positive cells (0–4), yielding a total score ranging from 0 to 12. Representative IRS scores for selected images were as follows: **(A1)**, 0; **(A2)**, 1; **(D2)**, 6; and **(C3)**, 12. Samples with IRS ≥ 3 were defined as high *ORC6* expression, whereas samples with IRS < 3 were defined as low *ORC6* expression; the median IRS was 3. Brown DAB staining indicates positive *ORC6* expression, and blue staining indicates hematoxylin counterstaining. Original magnification, ×20.

Samples were classified into high- or low-expression groups based on the median IRS score (cutoff = 3), with IRS ≥ 3 defined as high *ORC6* expression and IRS < 3 defined as low *ORC6* expression (**Figure 6**). Of the 163 clinical specimens, 71 cases (43.6%) were classified as high-expression and 92 cases (56.4%) as low-expression. Analysis of clinicopathological characteristics (**Table 1**) revealed a significant positive correlation between *ORC6* expression levels and pathological grading (*P* = 7.7010^-^³). No significant differences were observed between the low- and high-*ORC6* expression groups in age distribution, overall HPV infection status, HPV infection type, HPV16 status, or HPV18 status. However, HPV58 positivity was more frequent in the high-*ORC6* expression group than in the low-*ORC6* expression group (*P* = 0.043). The incidence rates of lymph node metastasis, lymphovascular invasion, and perineural invasion showed no statistical differences between the high- and low-expression groups (all *P*>0.05). These results indicate that high *ORC6* expression is primarily associated with the histological grade of cervical lesions rather than with invasive or metastatic clinicopathological features.

**Table 1:** Clinicopathological characteristics of the *ORC6* high- and low-expression groups.

Clinical features	Low expression (n=92)	High expression (n=71)	Total (n=163)	P value
Age				0.721
(Mean ± SD)	48.51±11.45	49.21±13.06	48.82±12.15	
Pathologic Grade				7.70×10^−3^
CIN I	21 (12.88%)	13 (7.98%)	34 (20.86%)	
CIN II-III	23 (14.11%)	36 (22.09%)	59 (36.20%)	
Moderately to well-differentiated	40 (24.54%)	18 (11.04%)	58 (35.58%)	
Poorly differentiated	8 (4.91%)	4 (2.45%)	12 (7.36%)	
HPV infection				0.382
HPV negative	13 (14.13%)	6 (8.45%)	19 (11.66%)	
HPV positive	79 (85.87%)	65 (91.55%)	144 (88.34%)	
HPV types^a^				0.716
High-risk only	57 (72.15%)	46 (70.77%)	103 (71.53%)	
Low-risk only	12 (15.19%)	8 (12.31%)	20 (13.89%)	
Co-infection	10 (12.66%)	11 (16.92%)	21 (14.58%)	
Most common HPV types				
HPV 16	32 (34.78%)	27 (38.03%)	59 (36.20%)	0.791
HPV 18	6 (6.52%)	4 (5.63%)	10 (6.13%)	1.000^b^
HPV 58	9 (9.78%)	16 (22.54%)	25 (15.34%)	0.043
Lymph node metastasis				1.000
Yes	9 (5.52%)	7 (4.29%)	16 (9.82%)	
No	83 (50.92%)	64 (39.26%)	147 (90.18%)	
Vascular invasion				0.112
Yes	22 (13.50%)	9 (5.52%)	31 (19.02%)	
No	70 (42.94%)	62 (38.04%)	132 (80.98%)	
Perineural invasion				0.333
Yes	7 (4.29%)	2 (1.23%)	9 (5.52%)	
No	85 (52.15%)	69 (42.33%)	154 (94.48%)	

^a^Percentages for HPV types were calculated among HPV-positive cases only.^b^Fisher's exact test.

**Figure 7:**
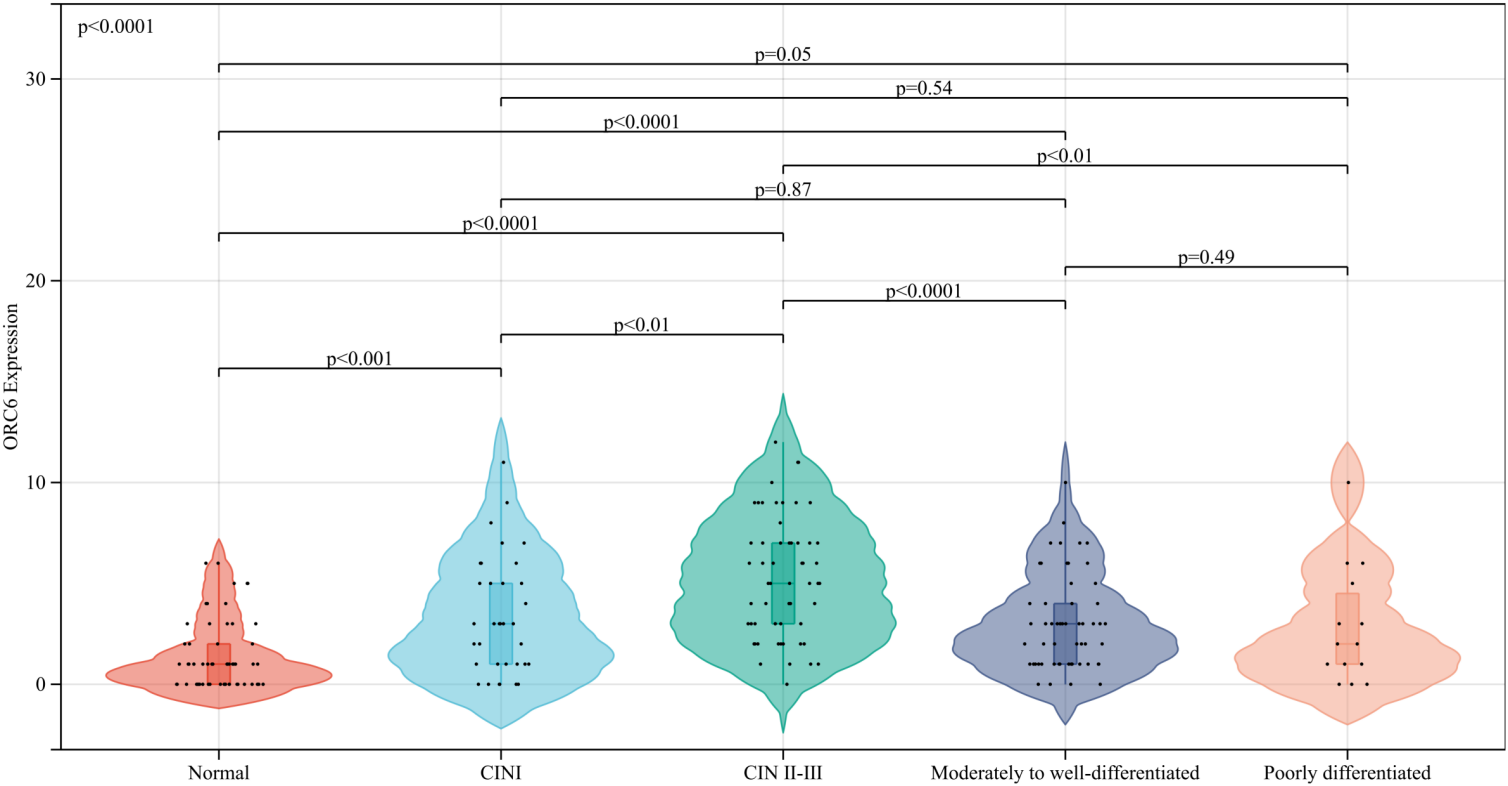
Distribution of *ORC6* immunoreactive scores across different pathological stages of cervical lesions.

To further evaluate the dynamic changes in *ORC6* expression across the progression of cervical lesions, we analyzed IRS scores as continuous variables (**Figure 7**). Compared with normal cervical tissues, *ORC6* expression was significantly increased in both CIN lesions and invasive cervical carcinoma (*P*<0.05).Notably, a significant upregulation of *ORC6* levels was observed during the progression from low-grade intraepithelial neoplasia (CIN I) to high-grade intraepithelial neoplasia (CIN II-III) (*P*<0.001). In contrast, no significant differences were observed between CIN II-III and moderate-to-well differentiated or poorly differentiated invasive carcinomas (all *P*>0.05). Likewise, no statistically significant difference was found between the CIN I group and the invasive cancer group (*P=*0.87 and *P=*0.64, respectively). Overall, *ORC6* expression showed a marked increase during the transition from CIN I to CIN II–III, whereas no further increase was observed after progression to invasive carcinoma. These findings suggest that *ORC6* overexpression is closely associated with the progression from low-grade to high-grade cervical intraepithelial lesions and may have potential value as an auxiliary biomarker for distinguishing LSIL from HSIL.

## 4 Discussion

Cervical cancer remains one of the primary malignancies threatening women’s health globally (1). Persistent high-risk HPV infection is the key etiological factor underlying cervical carcinogenesis, but the transition from HPV infection to high-grade dysplasia and invasive cancer is highly heterogeneous (2, 16, 17). Therefore, biomarkers that reflect the progression of HPV-related cervical lesions may help improve early risk stratification and pathological assessment. In the present study, we found that *ORC6* was significantly upregulated in cervical cancer and, notably, showed a marked increase during the transition from CIN I to CIN II–III. Elevated *ORC6* expression was also associated with cell-cycle and proliferation-related transcriptional programs, together with a distinct immune infiltration pattern. These findings suggest that *ORC6* may serve as a potential auxiliary biomarker for the identification of HPV-related high-grade cervical lesions.

*ORC6* is an essential component of DNA replication initiation, and our analyses showed that elevated *ORC6* expression in cervical cancer was associated with transcriptional features related to cell-cycle progression, DNA replication, and genome maintenance. The *ORC6*-high group showed coordinated expression changes in multiple proliferation-related genes, and GSEA demonstrated positive enrichment of hallmark pathways such as E2F targets, G2M checkpoint, and DNA repair. Similar associations between *ORC6* overexpression and proliferative activity have been reported in several tumor types. Pan et al. found that *ORC6* was associated with enhanced DNA replication signals in clear cell renal cell carcinoma, correlating with poor prognosis (18). Notably, Ibrahim et al. demonstrated that a gene signature composed of *ORC6* and SKP2 had prognostic value in breast cancer, outperforming traditional mitotic scores and Ki-67 across multiple cohorts (19). In addition, elevated *ORC6* expression has been associated with malignant proliferation and adverse outcomes in hepatocellular carcinoma, non-small cell lung cancer, and glioma (20–22). It is well established that high-risk HPV E6/E7 proteins release the inhibition of the E2F-MYC-Cyclin/CDK pathway by inactivating p53 and pRb, thereby forcing cells into the S phase and triggering persistent replication stress (6, 23). In this context, our transcriptomic results are biologically plausible, as elevated *ORC6* expression was associated with enrichment of E2F targets, G2M checkpoint, and DNA repair pathways. Given the established role of *ORC6* in DNA replication initiation, these findings suggest that *ORC6* overexpression may be linked to HPV-related cell-cycle dysregulation and increased replication demand during cervical lesion progression. Therefore, rather than demonstrating a direct causal role, our data support a potential association between *ORC6* upregulation and the transition from low-grade to high-grade cervical lesions under persistent HPV-driven proliferative stress.

Beyond cell-cycle dysregulation, failure of host immune surveillance is another important feature in the progression from persistent HPV infection to cervical cancer. Previous studies have suggested that *ORC6* expression may be linked to immune-related alterations in tumors. For example, Lin et al. reported that high *ORC6* expression was associated with immunoregulatory genes, including TGFBR1 and PD-L1, as well as regulatory T-cell infiltration across multiple tumor types (10). Another multi-omics study also showed that *ORC6* expression was associated with the upregulation of several immune checkpoint-related molecules (11). In our study, CIBERSORT analysis showed that elevated *ORC6* expression was associated with a distinct immune infiltration pattern, characterized by increased M0 macrophages and reduced activated NK cells, resting mast cells, and other immune cell subsets. In the context of cervical lesions, this finding is of potential relevance because NK cells and CD8+ T cells are important components of the host immune response against HPV-infected cells (24). In addition, the enrichment of M0 macrophages may indicate a tumor context associated with altered myeloid cell composition (13). Taken together, these findings suggest that elevated *ORC6* expression is associated with an immune infiltration pattern that may be relevant to impaired immune surveillance in HPV-related cervical lesions. However, given that our immune analyses were based on transcriptomic deconvolution and correlation analysis, further functional studies are needed to clarify whether *ORC6* directly contributes to immune modulation during HPV-related cervical lesion progression.

Clinically, our immunohistochemical analysis revealed a distinct expression pattern of *ORC6* across the progression of cervical lesions. *ORC6* expression increased markedly during the transition from CIN I to CIN II–III but did not show a further significant increase after progression to invasive carcinoma, instead remaining at a relatively high level. Consistent with this pattern, *ORC6* expression was significantly associated with pathological grade, but not with lymph node metastasis, vascular invasion, perineural invasion, or overall survival. These findings indicate that ORC6 overexpression may be more closely associated with the development of high-grade intraepithelial lesions than with later events of tumor progression. From a molecular pathological perspective, CIN II–III represents a critical stage in cervical carcinogenesis and is widely regarded as the stage most closely associated with progression risk. At this stage, persistent expression of high-risk HPV E6/E7 is known to be associated with genomic instability and marked cell-cycle dysregulation (6, 25). In our study, elevated *ORC6* expression was most prominent at this pathological stage, supporting the possibility that *ORC6* upregulation is linked to HPV-related replication stress and increased proliferative demand in precancerous lesions. Taken together, the potential clinical value of *ORC6* may lie primarily in auxiliary early warning and precancerous lesion grading, rather than in the evaluation of late-stage prognosis.

In conclusion, by integrating bioinformatic analyses with clinical validation, this study showed that *ORC6* is upregulated in cervical cancer and HPV-related cervical lesions, with a marked increase during the transition from CIN I to CIN II–III. Elevated *ORC6* expression was associated with transcriptional programs related to cell-cycle progression and DNA replication, as well as a distinct immune infiltration pattern. These findings suggest that *ORC6* may have potential value as an auxiliary biomarker for the identification and early stratification of HSIL.

Nevertheless, several limitations of the present study should be noted. The clinical cohort was retrospective and derived from a single center, and survival analysis was performed only in the TCGA cohort. In addition, the biological role of *ORC6* in cervical lesion progression was inferred mainly from transcriptomic analysis and immunohistochemical validation, and no *in vitro* or *in vivo* functional experiments were conducted. Therefore, further multi-center studies with larger cohorts and mechanistic investigations are warranted to better define the clinical utility and biological significance of *ORC6* in HPV-related cervical lesion progression.

## Data Availability

Publicly available datasets were analyzed in this study. These data can be found at the UCSC Xena platform (https://xenabrowser.net/datapages/): (1) transcriptomic data from the UCSC Toil RNA-seq Recompute Compendium (cohort: TCGA TARGET GTEx; dataset ID: TcgaTargetGtex_rsem_expected_count); (2) survival data from the TCGA-CDR curated dataset (cohort: TCGA Cervical Cancer (CESC); dataset ID:survival/CESC_survival.txt). The original contributions presented in the study are included in the article material, further inquiries can be directed to the corresponding author.
